# Adhesion of the probiotic bacterium *Lactobacillus plantarum *299v onto the gut mucosa in critically ill patients: a randomised open trial

**DOI:** 10.1186/cc3522

**Published:** 2005-04-28

**Authors:** Bengt Klarin, Marie-Louise Johansson, Göran Molin, Anders Larsson, Bengt Jeppsson

**Affiliations:** 1 Consultant, Assistant Professor Department of Anaesthesiology & Intensive Care, University Hospital, Lund, Sweden; 2 Research manager, Probi AB, Ideon, Lund, Sweden; 3 Professor, Laboratory of Food Hygiene, Lund University, Lund, Sweden; 4 Professor, Department of Anaesthesiology, Aalborg University Hospital, Aalborg, Denmark; 5 Professor, Department of Surgery, University Hospital, Malmö, Sweden

## Abstract

**Introduction:**

To achieve any possible positive effect on the intestinal mucosa cells it is important that probiotics adhere tightly onto the intestinal mucosa. It has been shown in healthy volunteers that *Lactobacillus plantarum *299v (Lp 299v) (DSM 9843), a probiotic bacterium, given orally in a fermented oatmeal formula adheres onto the intestinal mucosa, but whether this also occurs in critically ill patients is unknown.

**Methods:**

After randomisation, nine enterally fed, critically ill patients treated with broad-spectrum antibiotics received an oatmeal formula fermented with Lp 299v throughout their stay in the intensive care unit; eight patients served as controls. Biopsies of the rectal mucosa were made at admission and then twice a week, and the biopsies were analysed blindly.

**Results:**

Four patients in the control group were colonised with Lp 299v at admission but thereafter all their biopsies were negative (Lp 299v is an ingredient in a common functional food, ProViva^®^, in Sweden). Of the treated patients none was colonised at admission but three patients had Lp 299v adhered on the mucosa from the second or third biopsy and in the following samples.

**Conclusion:**

This study shows that Lp 299v could survive the passage from the stomach to the rectum and was able adhere onto the rectal mucosa also in critically ill, antibiotic-treated patients.

## Introduction

In critical illness, the intestine has been indicted as a source of pathogens sustaining the inflammatory response initiating or maintaining multiple organ failure. Various interventions have therefore been proposed to limit the growth of putatively causative pathogens in the gut; for example, selective intraluminal eradication of facultative aerobic Gram-negative bacteria – selective digestive decontamination. Indeed, selective digestive decontamination reduces the infection rate, especially in the respiratory tract [1]. Although a meta-analysis [2] and a recent study in critically ill patients [3] suggest a decreased mortality using selective digestive decontamination, there is a risk of emergence of multiresistant bacteria by the high antibiotic load.

Another method, potentially more beneficial for the microbiological environment, to reduce growth of pathogens in the gut is the administration of probiotics – lactobacilli and bifidobacteria [4]. Intestinal permeability is increased during critical illness, particularly after burns, major trauma and sepsis [5-7], and bacterial translocation has been demonstrated in patients with bowel obstruction [8,9]. The administration of probiotic *Lactobacillus *strains in animal experiments has been associated with reduced bacterial translocation and intestinal inflammation [10,11].

The strain *Lactobacillus plantarum *299v (Lp 299v) has excellent adherence characteristics using the mannose binding sites on the mucosal cells [12]. In fact, in healthy volunteers oral administration of Lp 299v produced adherence onto and colonisation of the rectal mucosa and remained viable, verified by biopsies, for more than 11 days after end of administration [13]. The positive effects might be due to the lactobacilli fermenting nutritional carbohydrates and fibres to the preferred substrates for enterocytes – the short chain fatty acids. However, the mannose binding adhesion of Lp 299v [12] and the ability for Lp 299v to adhere to the intestinal mucosa are a possible basis for exclusion of other bacteria from adhering, thus preventing translocation. Furthermore, Lp 299v has been shown to stimulate the mucin-production in HT-29 cells [14,15]. To have beneficial effects, however, the lactobacilli should survive and adhere to the gut wall in sufficient numbers. Lp 299v is sensitive to several of the commonly used antibiotics (e.g. ampicillin, erythromycin, clindamycin, and trimethoprim/sulphamethoxaxol). In addition, the decreased gut motility often seen in critical illness might influence the transport of Lp 299v down to the lower gastrointestinal tract. Whether Lp 299v survives and adheres to the mucosa in the lower gastrointestinal tract in critically ill patients is therefore uncertain.

The primary aim of this pilot study was to examine this survival and adherence by obtaining rectal biopsies from critically ill, antibiotic-treated patients given Lp 299v enterally. The secondary aims were to evaluate the influence on the main groups of bacteria in the gut and explore the side effects of the treatment and to evaluate how the given product was tolerated when given to critically ill patients.

## Materials and methods

The present study was approved by the Human Ethics Committee at Lund University and was performed in compliance with the Helsinki Declaration. Informed consent was obtained from the patient or from the next of kin. The study was performed in the general intensive care unit (ICU) (nine beds) at Lund University Hospital.

The inclusion criteria were that the patient should be 18 years or older, should be critically ill (defined by a presumed need of intensive care for 3 days or more), should tolerate enteral feeding, should have no significant coagulation disorder or thrombocytopenia, and should have an indication for broad-spectrum antibiotics.

After inclusion (which was made within 12 hours after admission), randomisation was performed with sealed envelopes. Enteral nutrition was started within 24 hours after admission to the ICU. Nine patients (treatment group) were given the test solution in addition to the enteral formula, and eight patients (controls) received the enteral formula alone (Nutrodrip Standard, Nutrodrip Fiber, or Impact; Novartis AG, Basel, Switzerland)

The test solution consisted of a fermented oatmeal formula containing 10^9 ^colony-forming units/ml Lp 299v (Probi AB, Lund, Sweden and Skånemejerier AB, Malmö, Sweden). The formula was given through a nasogastric catheter every 6 hours. The two first patients in the treatment group were given 50 ml portions throughout their study period but, due to bowel distension, the dose was adjusted in the other six patients to 50 ml test solution every 6 hours for 3 days and then 25 ml every 6 hours throughout the rest of their stay in the ICU.

All patients received prokinetic agents – metoclopramid (Primperan; Sanofi, Paris, France), cisapride (Prepulsid; Janssen-Cilag, Beerse, Belgium and sodium picosulphate (Laxoberal; Boehringer Ingelheim, Ingelheim, Germany).

Biopsies from the rectal mucosa were taken in both groups on the admission day and thereafter twice a week. The first biopsy from patients in the treatment group was taken before the administration of bacteria. Administration of enteral nutrition was started as soon as the patients' circulatory and respiratory functions had been stabilised and in all patients before 24 hours after admission. Biopsies were sent blinded for analysis to the laboratory.

### Analysis of the biopsies

The pieces of tissue were washed three times in a solution (0.9% NaCl, 0.1% peptone, 0.1% Tween, and 0.02% cysteine) before dilution and inoculation. Viable counts were obtained from Rogosa agar (Oxoid; Basingstoke, Hampshire, England) incubated anaerobically at 37°C for 3 days for the enumeration of lactobacilli, from Violet Red Bile Glucose agar (Oxoid) incubated aerobically at 37°C for 24 hours for the enumeration of *Enterobacteriaceae*, and from perfringens agar base (Oxoid) + TSC selective supplement (Oxoid) incubated anaerobically at 37°C for 3 days (sulphite reducing clostridia). Colonies suspected to be Lp 299v on the Rogosa agar plates (large, creamy, white–yellowish and somewhat irregular) were counted. Representative colonies were picked, purified on Rogosa agar and were identified by Randomly Amplified Polymorphic DNA typing [16].

### Clinical routine cultures

Specimens from blood, urine and tracheal secretion, from wounds and from other relevant locations were sent for culture weekly or when clinically indicated. Tips from central venous catheters and occasionally, on suspicion of infection, arterial lines were sent for culture at removal.

The specimens were cultured and analysed at the Department of Clinical Microbiology, Lund University Hospital, according to clinical routines.

### Chemistry

Blood gases were analysed in the ICU and other routine experiments were performed at the Clinical Chemistry Laboratory, Lund University Hospital.

### Statistics

The proportions of conversion of bacterial adherence to the mucosa were analysed with the chi-square test (2 × 3 table) (Statview; SAS institute Inc., Cary, NC, USA). Differences in chemistry and bacterial counts of the main groups of bacteria were analysed with the Student *t *test (Statistica 6.0; Statsoft, Tulsa, OK, USA). *P *< 0.05 was considered significant. The results are presented as the median and range unless otherwise indicated.

## Results

All patients tolerated total or partial enteral feeding, and from day 2 the patients received at least 25% of the calculated daily nutritional needs via the enteral route. Supplementary nutrition was given parenterally.

Patients in the treatment group were older than the controls (median 70.9 [38–85] years versus 57.5 [34–76] years). There were no differences in the Acute Pathophysiology and Chronic Health Evaluation II score (17 [13–29] and 19 [14–36] for the treatment and control groups, respectively) in the days on a ventilator, in the median length of stay in the ICU (12 [4–37] days versus 11 [4–49] days), in hospital mortality (two patients died in each group) or in 6-month mortality (all patients discharged from the hospital survived) between the groups (Table 1).

All the patients were treated with broad-spectrum antibiotics, mainly imipenem and cefuroxime (Table 2), in consensus with a consultant physician from the Department of Infectious Diseases and according to results from previous cultures. In two patients, one from each group (patients 7 and 9), only one biopsy (before the start of the treatment) was obtained due to short stay; hence, these patients were excluded from the study. The calculations are thus based on eight patients in the treatment group and seven patients in the control group.

C-reactive protein was similar in the two groups throughout the study. The leukocyte count tended initially to be higher in the treatment group, but after day 5 the leukocyte count was lower in the treatment group (*P *= 0.036 on day 6). There was no difference in the other routine chemistry.

After the adjustment of the dose of the test solution the enteral solutions were well tolerated. There was no difference in the incidence of diarrhoea or gas bloating between the two groups.

### Cultures of biopsies and colonisation of Lp 299v

There was no significant bleeding or other side-effects after the biopsies in any patient.

The number of analyses of biopsies in the treatment group and in the control group were two analyses in six patients (three patients and three patients, respectively), three analyses in four patients (two patients and two patients, respectively), four analyses in two patients (one patient and one patient, respectively) and five analyses in three patients (two patients and one patient, respectively). There was a difference (*P *= 0.029) of bacterial conversion in the biopsies between the groups. At the start of the study, four out of seven control patients were positive for Lp 299v on the first biopsy but Lp 299v was not detectable in subsequent biopsies. In the treatment group, no patient was positive at admission, but two patients converted to positive culture for Lp 299v on the second biopsy and a third patient converted from the third biopsy. The successive tests remained positive in these three patients.

All patients received two or more doses of antibiotics before inclusion and the first biopsy. Five patients had been treated with antibiotics for more than 24 hours (3 days–3 weeks) before ICU admission. The antibiotics used before and during the study and the findings of Lp 299v from the biopsies are depicted in Table 2.

**Figure 1:**
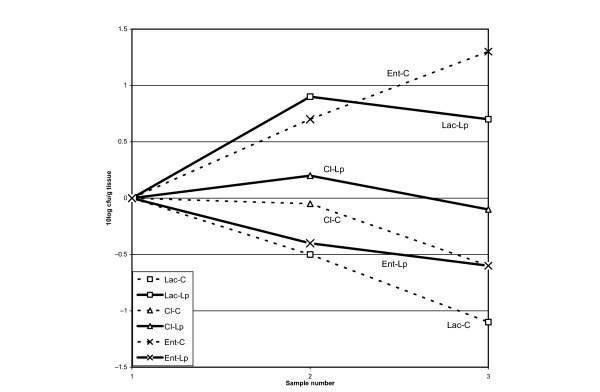
Changes of bacterial counts from rectal biopsies (means): comparisons with the initial sample. The *Enterobacteriaceae *(Ent) species show a 10-fold increase in mean values in the control (-C) group while *Lactobacillus *(Lac) decrease 10-fold. In contrast, in the treatment group (-Lp) *Lactobacillus *(Lac) increase and *Enterobacteriaceae *decrease. Sulphite reducing clostridia (Cl) decrease in the control group. cfu, colony-forming units.

The numbers of *Lactobacillus *increased in treated patients while there was a tendency for a reduction in the controls (*P *= 0.061) (samples from the second biopsies). We could not discern any statistical differences between the groups regarding *Enterobacteriaceae *or sulphite reducing clostridia (Fig. 1), although the mean values of *Enterobacteriaceae *increased in the control group and decreased in the treatment group (*P *= 0.27 comparing samples from the second round of samples).

From the 15 patients who completed the study, 240 cultures were performed from inclusion until 36 hours after transfer to other units. Fifty-eight (24%) of these cultures were positive (Table 3). In blood, five out of 32 cultures showed bacterial growth in the control group whereas none of 30 cultures in the treatment group had bacterial growth. In the treatment group blood cultures were taken from five out of the eight patients, and blood cultures were taken from five of seven patients in the control group. The positive cultures came from three patients. In patient 3 we found two different strains of coagulase-negative *Staphylococcus*. The samples were taken the same day but at different occasions. In patient 8 different enteric bacteria were found on two occasions, days apart. The fifth finding was a coagulase-negative *Staphylococcus *from patient 11. Findings were more equal in cultures from other sites.

The species found from the blood, the catheter tips, the tracheal secretions, and the urine results are presented in Table 4.

## Discussion

This pilot study shows that Lp 299v administered to critically ill, antibiotic-treated patients can survive and colonise the gut mucosa, and that repeated administration of the bacteria is necessary to obtain this effect.

The commercial market for probiotics today is worth about €6 billion, and the European Union has invested more than €15 million in studies of probiotics, but very few results have so far emerged [17]. Probiotics have been proposed to be beneficial for the gut as well as to decrease the risk of superinfections and the development of gastrointestinal malignancies, and to have positive effects on the immune system. However, although animal experiments have shown some beneficial effects [10,11,18], very little is proven in humans. One reason for this could be that some of the proposed probiotics have no effect; even if the bacterium is 'friendly' or harmless but it does not adhere closely to the intestinal mucosa, it is probably not beneficial for the mucosal cells.

Manipulation of the gut flora by stimulating certain species, as opposed to the prevalent therapy today of suppression with antibiotics, may be a possible measure to prevent or reduce the frequency of secondary infections in severely ill patients.

*Lactobacillus *is an important component of the mucosa-associated flora in humans, but it is not the predominating genus on the colonic mucosa. Other genera are present at the same level or at higher levels [18-20]. Lactobacilli have been claimed to have several therapeutic functions; for example, to prevent diarrhoea, to reduce translocation and to exert immune modulation. Lp 299v is obtained from human colonic mucosa, and this particular strain possesses an excellent ability to establish itself and to adhere to the mucosa [12,13,21]. This is the first time it has been shown that a bacteria like this can be established on the gastrointestinal tract mucosa in critically ill patients.

We have previously shown that Lp 299v does adhere to the mucosa in about 40% of healthy volunteers [13]. In a study on healthy volunteers where 19 different strains of *Lactobacillus *were given in fermented oatmeal soup, only five strains were retrieved from any of the 13 participants either from jejunal or rectal mucosal biopsies [13]. Biopsies were taken before administration and on day 1 and day 11 after administration had ended. On day 1 post treatment, Lp 299v or *Lactobacillus plantarum *299 (similar to Lp 299v and hence analysed as the pair) was found on rectal biopsies from four of the 13 volunteers and, remarkably, on biopsies from six participants on day 11 post treatment. By comparing this with our results where three out of eight treated patients turned from negative to positive on these cultures for Lp 299v, we conclude that the frequency of establishment is about the same as in healthy non-antibiotic-treated volunteers. Why all volunteers or patients did not convert to detectable levels (2 × 10^3^/g tissue) probably has multifactorial explanations, including genetic factors and original microbiotic flora.

In the present pilot study on critically ill patients, however, antibiotics did not seem to be an important factor in preventing survival and mucosal adherence of Lp 299v when distributed enterally.

Our study was not powered to analyse gastrointestinal or systemic effects but there is a demand for such studies because probiotics are now routinely used in many ICUs without any strong scientific proof of beneficial effects. There are, however, some small studies indicating positive effects. In a study by Oláh and colleagues, 22 patients with acute pancreatitis were given *Lactobacillus plantarum *299 and 23 patients were given only the oatmeal formula (with heat-inactivated bacteria) [22]. The authors found a significant decrease in episodes of sepsis and pancreatic abscesses in the treated patients.

Rayes and colleagues randomised 95 liver transplantation recipients into three groups, all feed enterally [23]. One group received standard enteral formula plus selective bowel decontamination, a second group received fibre-containing formula plus *Lactobacillus plantarum *299, and the third group received the same regimen as the second group but the lactobacilli had been heat-killed. The infection rate was reduced by 35% in the group given active bacilli compared with the group given standard formula or heat-killed bacteria. On the other hand, in another study by the same research group there was no difference in the infection rate between surgical patients that received active *Lactobacillus plantarum *299 and patients who received heat-killed lactobacilli [24].

In addition, two studies by McNaught and colleagues have not shown any positive effect of probiotics in patients undergoing major surgery [25,26]. It should be pointed out, however, that the amount of bacteria administered in the three latter studies was probably inadequate; the daily doses of bacteria were only 5–10% of the daily dose administered in our study. Which dose is sufficient and whether probiotics have any positive effects in critically ill patients are thus still inconclusive factors.

The increase of lactobacilli on the rectal mucosa is most probably due to the administration of relatively large numbers of the study bacteria. All other changes that occurred in the amount of bacteria were not statistically significant. It is possible, however, that this is only due to the low power of the study and does not indicate a biological fact. Mean values of *Enterobacteriaceae *showed dispersing values for treated patients and control patients, and this might imply that the enterally added *Lactobacillus *changes the gut milieu so that the growth of pathogenic bacteria is inhibited.

Interestingly, the result from other cultures showed no growth of bacteria in blood cultures from the treated patients in contrast to the control group showing 15% positive cultures. This could indicate an effect of Lp 299v on the mucosal barrier, or on the immune system, as shown in the studies on *Lactobacillus plantarum *299 on pancreatitis transplant patients and liver transplant patients [23,24].

Our study has several limitations. First, only a few patients were included. We wanted to study as low a number of patients as possible, due to the inherent risks with rectal biopsies, but still wanted to be able to assess whether adherence of Lp 299v could occur in critical illness. An experienced surgeon performed the biopsies and we used very strict inclusion criteria in order to increase the safety of the procedure and to prevent harmful side-effects. Indeed, we had no complications.

Second, four patients in the control group already had growth of Lp 299v on rectal biopsies when entering the study. This is most probably due to the fact that this bacteria is commercially available as part of a probiotic fruit beverage (made from the same base as our study product) in Sweden and is widely consumed by the population. In addition, since the organism used was originally harvested from human mucosa [27], our findings might be explained by the natural occurrence of the bacteria. The bacteria, however, were not identified on the subsequent biopsies in these patients, suggesting that regular administration is necessary to maintain the adhesion onto the mucosa.

Third, the statistics used could be questioned. Nevertheless, there is no reasonable explanation for the conversion from no adherence to adherence of the Lp 299v onto the mucosa other than the enteral administration of this strain *per se*.

Finally, in the patients in whom we did not find any bacterial adhesion on the rectal mucosa, we cannot exclude that that the bacteria adhered onto the mucosa at other parts of the gastrointestinal tract.

## Conclusion

In conclusion, this pilot study shows that enteral administration of Lp 299v is feasible in the intensive care setting. The study also shows that this bacterium can survive transport in the gastrointestinal tract and seems to colonise the gut mucosa, as assessed from rectal biopsies, in critically ill patients treated with broad-spectrum antibiotics.

## Key messages

• 	The probiotic bacteria *Lactobacillus plantarum 299v*, given enterally to critically ill patients on antibiotic therapy survives the passage through the gastrointestinal tract and has the ability to colonize the rectal mucosa

• 	It is necessary to administer Lp 299v daily when patients are on antibiotic therapy.

• 	We saw no adverse effects and the study product containing oatmeal soup was well tolerated.

• 	Administration increases the number of lactobacilli and reduces the number of *Enterobacteriaceae*.

• 	The absence of positive cultures in the treatment group indicates that Lp 299v may have an effect on the mucosal barrier or even have a positive impact on the immune system.

## Abbreviations

ICU = intensive care unit; Lp 299v = *Lactobaccilus plantarum *299v.

## Competing interests

BJ, GM and M-LJ are shareholders in Probi AB. Probi AB provided the study product.

## Authors' contributions

BK, the primary investigator, was active in study planning, performed all beside work apart from the biopsies, handled the primary data and some of the statistical work, and prepared and finalised the manuscript together with GM, AL and BJ. M-LJ was active in the planning and practical performance of the study, and performed some of the statistical analysis. AL was involved in the study layout, performed some of the statistical analysis and was active in preparing the manuscript. GM contributed to analyses of the results from the bacterial cultures and to finalising the manuscript. BJ participated actively in the planning of the study and in the preparation of the manuscript.

## Figures and Tables

**Table 1:** Patient characteristics

Patient	Age (years), gender	Diagnosis at admission	APACHE II score	Length of stay in ICU (days)
Treatment group				
2	38, female	Pneumonia	13	14
4	63, male	Gun shot wound	15	10
5	52, female	Respiratory insufficiency	15	15
10	69, female	Pancreatitis	17	37
12	84, male	Pneumonia	24	4
14*	84, female	Pneumonia	23	10
15^†^	72, male	Respiratory insufficiency	29	20
17	77, female	Sepsis	17	4
Control group				
1	33, male	Multi-trauma	14	5
3	57, female	Pancreatitis	19	20
6	57, male	Pneumonia	15	11
8^†^	61, male	Septic arthritis	24	49
11^†^	60, male	Retropharyngeal abscess	19	19
13	76, male	Respiratory insufficiency	36	4
16	56, female	Sepsis	16	7

APACHE, Acute Pathophysiology and Chronic Health Evaluation. *Died in the hospital after the intensive care unit (ICU). ^†^Died in the ICU.

**Table 2:** Identification of *Lactobacillus plantarum *299v (Lp 299v) from biopsies and the antibiotics used

Patient	Lp 299v, first biopsy	Lp 299v, later biopsies	Antibiotics prior to ICU admission (≤ 12 days if not specified)	Antibiotics in ICU before first biopsy	Antibiotics in ICU (during biopsy period)
Treatment group					
2	No	Yes	Erythromycin	Erythromycin + imipenem	Erythromycin + imipenem
4	No	No	Cefuroxime	Cefuroxime	1 Imipenem, 2 +metronidazol
5	No	No	Cefadroxile, 10 days	Cefadroxile	1 Cefuroxime, 2 meropenem
10	No	No	Cefuroxime, 3 days	Imipenem	1 Imipenem, 2 +metronidazol
12	No	No	No antibiotics	Imipenem	Imipenem
14	No	Yes	1 Metronidazol + cefotaxime/cefuroxime, 2 -metronidazol, 3 -cefotaxime/cefuroxime; + imipenem; 12 days in total	Imipenem	Imipenem
15	No	Yes	Ciprofloxacin + two doses metronidazol (rectally)	Ceftazidime	1 Ceftazidime, 2 +metronidazol
17	No	No	Cefuroxime	Imipenem	Imipenem
Control group					
1	No	No	Cloxacillin	1 Cloxacillin, 2 cefuroxime	1 Cefuroxime, 2 +metronidazol
3	Yes	No	Imipenem	Imipenem	1 Imipenem, 2 +metronidazol
6	Yes	No	1 Penicillin G, 2 erythromycin, 3 +netilmicin, 4 cefotaxime (-netilmicin, -erythromycin), 5 erythromycin, 6 imipenem; 3 weeks in total	Imipenem	Imipenem
8	Yes	No	Penicillin G	Imipenem	1 Imipenem, 2 +clindamycin, 3 -clindamycin, +metronidazol, 4 vancomycin+ ciprofloxacin
11	No	No	Metronidazol and cefuroxime	Metronidazol and cefuroxime	1 metronidazol + cefuroxime, 2 +isoniazid, 3 +rifampicin, 4 -(1, 2, 3), +imipenem
13	Yes	No	1 PenicillinV, 2 cefuroxime; 6 days in total	Cefuroxime	Cefuroxime
16	No	No	Cefuroxime	Cefuroxime	1 Cefuroxime, 2 penicillin G

Figures indicate the order in which antibiotics were been given (and changed). +, added medication; -, withdrawn medication. ICU, intensive care unit.

**Table 3:** Number of cultures

Type of culture	Control group			Treatment group			Fisher's exact test
		
	*n*	Positive *n*	Number of patients with positive cultures	*n*	Positive *n*	Number of patients with positive cultures	
All	122	25	5/7	118	33	6/8	NS
Blood	32	5	3/7 (3/5)	30	0	0/8 (0/5)	NS
Catheter tips	22	4	3/7 (3/4)	22	4	3/8 (3/6)	NS
Tracheal secretions	14	6	2/7 (2/6)	15	6	5/8 (5/6)	NS
Urine	19	1	1/7 (1/7)	18	4	2/8 2(/6)	NS

Figures in parentheses show the number of patients with positive cultures in relation to the number of patients from whom the respective type of culture were taken. In the treated group, five cultures were positive in the control group while no positive cultures were found in the treatment group. Due to the small numbers of patients (we performed statistics as participating patients and not as independent cultures), a significant difference was not reached (NS, not significant).

**Table 4:** Species found at different locations

Location	Control group	*Lactobacillus plantarum *299v group
Blood	Coagulase-negative *Staphylococcus*, 3	None
	*Enterococcus faecalis*, 1	
	*Pseudomonas aeruginosa*, 1	
Catheter tips	Coagulase-negative *Staphylococcus*, 3	Coagulase-negative *Staphylococcus*, 3
	*Enterococcus faecium*, 1	*Morganella morgani*, 1
	*Enterobacter cloacae*, 1	*Enterococcus faecalis*, 2 (1 scarce)
Tracheal secretions	*Klebsiella pneumoniae*, 3	*Escherichia coli*, 2
	*Pseudomonas aeruginosa*, 1	*Morganella morgani*, 1
	*Enterococcus faecalis*, 2	*Pseudomonas aeruginosa*, 1
		*Enterococcus faecium*, 1
		*Enterobacter cloacae*, 1
		*Candida albicans *(scarce), 1
		*Candida kefyr*, 1
Urine	*Pseudomonas aeruginosa*, 1	*Candida albicans *(scarce), 2
	*Enterococcus faecalis*, 1	*Candida tropicalis *(samples from one patient, same day but separated in time), 2

Main differences between the treatment and control groups are, besides no positive blood cultures, the more abundant findings of fungi. The growth of fungi in the treatment group (urine and tracheal secretions) might be due to less bacteria giving better conditions for the culturing of fungi.
